# Understanding If Differences in Salivary Flow Rate and Total Protein Content Triggered by Biological Factors (Sex and Age) Affect Aroma Perception and the Hedonic and Emotional Response of Wine Consumers

**DOI:** 10.3390/foods11193104

**Published:** 2022-10-05

**Authors:** Celia Criado, Carolina Muñoz-González, María Mora, Virginia Fernández-Ruíz, Carolina Chaya, María Angeles Pozo-Bayón

**Affiliations:** 1 Instituto de Investigación en Ciencias de la Alimentación (CIAL), Consejo Superior de Investigaciones Científicas-Universidad Autónoma de Madrid (CSIC-UAM), C/Nicolás Cabrera, 9, 28049 Madrid, Spain; 2 BCC Innovation, Centro Tecnológico en Gastronomía, Basque Culinary Center, 20009 Donostia-San Sebastián, Spain; 3 Basque Culinary Center, Faculty of Gastronomic Sciences, Mondragon Unibersitatea, 20009 Donostia-San Sebastián, Spain; 4 Departamento de Economía Agraria, Estadística y Gestión de Empresas, Escuela Técnica Superior de Ingeniería Agronómica, Alimentaria y de Biosistemas (ETSIAAB), Universidad Politécnica de Madrid, Avda. Complutense s/n, Ciudad Universitaria, 28040 Madrid, Spain; 5 Departamento Nutrición y Ciencia de los Alimentos, Universidad Complutense de Madrid, Plaza de Ramón y Cajal s/n, 28040 Madrid, Spain

**Keywords:** wine, oral physiology, saliva, aroma perception, consumer’s response, self-reported emotions

## Abstract

The relationship between oral physiology (e.g., salivary protein content) and aroma perception over wine consumption was investigated in previous work. However, the relationship between oral physiology and the consumer’s response to wine is unknown. Additionally, age–gender differences might affect oral physiology and, therefore, inter-individual differences in the consumer’s enjoyment of wine. The aim of this work was to study the effect of biological individual factors such as sex and age on salivary flow rate and total protein content, on wine retronasal aroma perception and on the hedonic and self-reported emotional consumer response to wine. Additionally, the relationship between saliva composition, aroma perception and liking was also assessed. Results showed that age and sex influenced saliva composition and aroma perception in wine consumers in the case of red but not white wines. Younger females reported higher aroma intensity of the black pepper descriptor compared to older females. Correlation analysis confirmed the relationship between salivary flow rate and aroma perception and between the salivary protein content and wine acceptability. The interactions between wine polyphenols–saliva–aroma that occurred in the mouth during the oral processing of wine might partially explain these results, although further research will be required to confirm this hypothesis.

## 1. Introduction

During wine consumption, saliva can play an important role in aroma perception. Through its multiple effects on odorant molecules (enzymatic metabolism, interaction with proteins, and dilution), saliva can modify oral aroma release and the type and quantity of odorant molecules that reach the olfactory receptors [1,2,3,4,5].

Salivary flow rate and saliva composition can also vary during lifespan [6,7,8,9]. Additionally, sex-related changes in saliva composition were reported [10]. Inter-individual differences in saliva composition and flow rate recently were related to dynamic changes in flavour perception during the full experience of tasting [11,12]. For instance, some characteristic wine aroma attributes (black pepper and smoky) were more intensely and for longer times retronasally perceived in senior individuals (over 55 years old) compared to young adults (18–35 years old). The better performance of the senior individuals was positively correlated with the content in salivary proteins and negatively with the salivary flow rate [13]. More recently, Pérez-Jiménez and collaborators [14] studied the differences in the quantity of in-mouth aroma release during wine tasting, depending on age–gender groups, showing a higher effect of age than gender. In this work, the oral release of certain types of volatiles (furanic compounds and C_13_ norisoprenoids) was higher in senior individuals compared to younger ones.

Since aroma represents one of the most important sensory attributes linked to consumer preference [15], differences in saliva composition due to biological factors such as age and sex might have an effect on wine acceptability.

In this sense, the preference for certain types of wine depending on the individual’s age was described in previous works [16,17]. Young people prefer fruitier wines while older individuals prefer more complex wines [18,19]. Nonetheless, to the authors’ knowledge, the relationship between changes in saliva composition as a consequence of intrinsic biological factors (e.g., age, sex) and wine preference was not tested with wine consumers.

Additionally, it is worth noting that assessing only wine acceptability might not be enough to explain the consumer’s response to wine, since emotional aspects are strongly linked to the sensory experience, also affecting the hedonic response [17,20,21,22,23]. Therefore, combining consumer acceptability and emotional response could provide a better input in order to understand the consumers’ response to wines [23].

Regarding the emotional response evoked by food, previous works have already shown correlation with age. For instance, younger adults reported more extreme scores on food-evoked emotions, whereas older adults (over 55 years old) gave more neutral scores [17,24,25,26]. Additionally, older adults reported higher scores in positive emotions than young adults [27,28,29]. In wines, a complex relationship between younger (18–35 years old) and senior (over 55 years old) consumers was observed: the seniors scored the positive emotions higher than the young adults, but more differences between wines were reported by the younger consumers [17].

Moreover, differences in the emotional response to wines between gender groups were established [17]. In this study, wines evoked higher ratings in positive emotions for men than for women, although women showed a better discrimination among different types of wine.

Considering the impact of saliva composition on aroma perception and the importance of this sensory modality on wine consumer preferences, the aim of this work was to study the effect of biological individual factors, such as sex and age, on salivary flow rate and total protein content, on wine retronasal aroma perception and on the consumer’s response to wine (hedonic and emotional). Overall, this study will provide the first input on the relationship between oral physiology (e.g., salivary composition) and the consumer’s response to wine, which is an important aspect of understanding the inter-individual differences in the consumer’s affective response to wine.

## 2. Materials and Methods

### 2.1. Wine Samples

Two commercial wines with distinctive sensory attributes were employed in this study: a Rioja (PDO) red oak aged wine, made in 2014 (84% Tempranillo, 9% Graciano, 5% Mazuelo, 2% Garnacha) with 14% ethanol (*v*/*v*), and a white wine from Rueda (PDO) made in 2018 from the Verdejo white variety with 13.5% ethanol (*v*/*v*). In previous sensory trials (performed one month before the consumer study) using a tasting panel, the two predominant aroma attributes, i.e., black pepper and pineapple, were noticed as the most characteristic of red and white wines, respectively.

Additionally, a neutral (minimally aromatic) commercial rosé wine, made from Tempranillo with 11.0% ethanol (*v*/*v*) (Cerro de la Cruz, Spain), was used as a warm-up sample.

### 2.2. Consumer Recruitment

Consumers were recruited using advertisements posted on campuses of participating universities, by email and through social networks (Twitter, Facebook). The inclusion criteria for the participation in the study were to be healthy and non-pregnant adult volunteers (18 years old) and regular wine consumers (they had to consume wine at least once a month). Additionally, they had to belong to one of the two specific age groups screened: young group (individuals between 18 and 35 years old) and senior group (individuals over 55 years old). In addition, all volunteers filled a food allergy screening document, including allergy/intolerance to wine or any of its components. The Bioethical Committee of the Spanish National Council of Research (CSIC) approved this study (CSIC, 2016). Before starting the study, the volunteers read and signed an informed consent form.

A total number of 103 volunteers participated in this study, in which not only sensory, but salivary parameters were collected. Participants were divided into two age groups: young (56.3%) and senior (46.7%) adults. Each age group was also balanced by sex. In this study, sex (biological) and gender (social) terms will be used indistinctively, since the entire consumer group identified their gender with their biological sex. The consumers were made up of 22.3% young females (26.38 ± 3.50 years old), 21.4% young males (26.13 ± 3.01 years old), 33.0% senior females (61.39 ± 5.02 years old) and 23.3% senior males (62.87 ± 5.26 years old). Independence of age and gender distribution of the consumers’ sample was verified by a Chi-Square test.

### 2.3. Saliva Collection and Characterization

Saliva collection was carried out immediately prior to the consumer test. One hour before saliva collection, volunteers were asked not to smoke, drink or eat. Stimulated saliva was collected directly in a tube (previously weighed). The participants were told to avoid swallowing during the saliva collection process. For saliva collection, subjects chewed a piece of Parafilm TM and spat their saliva out into the tube as many times as they wanted for 5 min. Salivary flow rate was calculated from the weight of saliva and expressed as mL/min. To avoid any possible deterioration of the samples before analysis, CaCl_2_ was added at a concentration of 30 mg/mL immediately after collection [30]. The saliva samples were homogenized by centrifugation at 15,000× *g* for 15 min at 4 °C, which facilitated biochemical analysis by removing excessive mucus and cells. Additionally, the centrifuged saliva samples were divided into aliquots and stored at −80 °C. All the analytical determinations were performed using centrifuged saliva.

For the analysis of the total protein content in saliva (TPC), the commercial kit Pierce™ BCA Protein Assay Kit (Pierce Thermo Scientific, Rockford, IL, USA) with bovine serum albumin as the calibration standard was used.

### 2.4. Consumer Tests

Consumer tests were performed at the sensory facilities of the Universidad Autónoma de Madrid (UAM) and the Universidad Politécnica de Madrid (UPM).

Wine samples (25 mL) from the red and white wines were served into standard wine tasting-glasses (20 cL) and presented to the consumers. To do so, the wine glasses were labelled with 3-digit random codes and simultaneously presented in a random order using a Complete Balanced Block design. Prior to testing the wines, an additional warm-up sample (rosé wine) was used to minimize the first position effect [31,32]. Mineral water (Nestle Aquarel, Barcelona, Spain) and breadsticks (ARO, Madrid, Spain) were provided as palate cleansers.

After tasting each wine, the consumers were asked to rate their liking using a 9-point hedonic scale (1 = dislike extremely; 9 = like extremely) after each tasting, whereafter the emotions elicited by each sample was rated. To measure the emotional response elicited by the wines, the lexicon II developed by Mora and collaborators [33] for Spanish consumers was used (Table S1). Wine consumers were asked to read all the terms associated with each emotion category and rated the intensity of the evoked feelings by each wine using an unstructured line scale, from ‘very low’ (0) to ‘very high’ (15). Prior to the test, the consumers were instructed to focus on their feelings associated with each specific sample and not to their general mood. The wine was tasted again to rate the perceived retronasal aroma intensity of black pepper and pineapple aroma using an unstructured line scale, from ‘very low’ (0) to ‘very high’ (15).

Data were collected with tablets using Compusense^®^ Cloud software (Compusense Inc., Guelph, ON, Canada).

### 2.5. Data Analysis

Analysis of variance models and Tukey tests were first carried out to analyse the effect of age and gender of the consumers on salivary parameters (salivary flow rate and TPC). Additionally, a two-way ANOVA with interaction was also performed to analyse the differences by age–gender groups on the perceived intensity of the two aroma attributes (black pepper and pineapple) in both wines. The dependent variables were the perceived aroma intensity of black pepper and pineapple, and the independent variables were the age–gender groups and the wines. The same procedure was used for liking and for each emotional category. Finally, Pearson’s correlation analysis was performed to check the relationship between salivary parameters, liking and aroma perception (black pepper and pineapple). Significant differences were determined with a significance level of 0.05. The statistical analyses were carried out using XLSTAT (XLSTAT Version 2019.01).

## 3. Results

### 3.1. Differences in Salivary Flow Rate and Total Protein Content (TPC) by Age–Gender Groups

To determine if saliva composition was different among age–gender groups of consumers, data from salivary flow rate and total protein content (TPC) was submitted to an ANOVA and Tukey test. Results are shown in Table 1.

As it can be seen, analysis of variance showed significant differences between age–gender groups for both salivary parameters (Table 1). For the salivary flow, the young–male group showed the highest value (1.57 ± 0.52 mL/min) while the lowest value corresponded to the senior–female group (1.10 ± 0.52 mL/min). Interestingly, the salivary flow rate of the males was higher than that of the females for both age groups (young and senior).

On the other hand, total protein content also showed differences between age groups, with the senior (senior–male, 1448.35 ± 549.03 mg/L; senior–female, 1483.44 ± 547.87 mg/L) presenting higher values than the young adults (young–male, 991.39 ± 539.96 mg/L; young–female, 1219.75 ± 553.24 mg/L). Moreover, the TPC values corresponding with the young females (1219.75 ± 553.24 mg/L) were slightly higher than those of the young males (991.39 ± 539.96 mg/L), although this difference was not statistically significant.

### 3.2. Differences in Retronasal Aroma Perception by Age–Gender Groups

**Figure 1:**
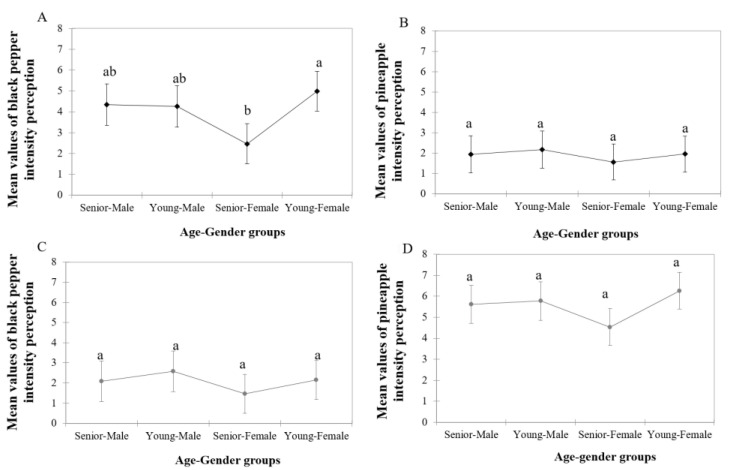
Effect of age–gender groups of consumers on the black pepper and pineapple aroma intensity retronasally perceived after tasting both wines: (**A**) black pepper intensity in red wine, (**B**) pineapple intensity in red wine, (**C**) black pepper intensity in white wine, (**D**) pineapple intensity in white wine. Different letters (a-b) show significant differences among age-gender groups from Tukey test (*p* < 0.05).

The intensity of the two selected aroma descriptors (black pepper and pineapple) was evaluated in the red and white wines considering the corresponding age–gender groups. Results from the one-way ANOVA are shown in Figure 1.

Significant differences were only observed between age–gender groups of consumers for black pepper intensity in the red wine (Figure 1). Tukey test showed that the significant differences (*p* < 0.05) in this attribute were between both female groups (Figure 1A). In fact, senior–females and young–females showed the lowest (2.46) and highest perception (4.98) of this attribute, respectively.

However, no significant differences were found between age–gender groups in the pineapple attribute. Additionally, as Figure 1 shows, the four groups of consumers rated the black pepper higher in the red wine than in the white wine. Similarly, both wines also differed in the intensity of the pineapple attribute, which was higher rated in the white than in the red wine.

### 3.3. Differences in Liking and Emotional Response to Wine by Age–Gender Groups

Regarding the differences in liking between age–gender groups, analysis of variance did not show a significant effect. In general, the highest values of liking for the white wine were noticed in the young–female group (6.82 ± 0.32) (Table 2), although these results were not statistically significant, and most of the groups rated the liking for this wine similarly. The scores of the red wine were also similar among age–gender groups, although the senior–male and young–female groups (6.45 ± 0.39, 6.44 ± 0.32, respectively) scored the wine slightly higher than the young–male group (5.96 ± 0.38).

**Figure 2:**
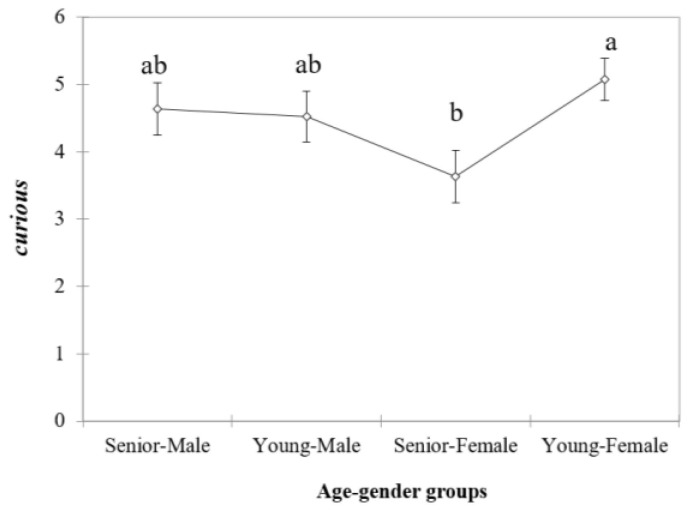
Mean scores (considering the two types of wines) of the emotional term “curious” according to the age–gender group. Different letters (a-b) show significant differences among age-gender groups from Tukey test (*p* < 0.05).

Interestingly, when applying Pearson correlation analysis, liking was significantly correlated with most of the emotional terms (Table 3). Most of the positive emotions (e.g., “sensitive”, “satisfied”, “lucky”, “cheerful”, “affectionate”, “joyful”, “anxious”, “nostalgic”, “satisfied”, “curious”, “fun”, “refreshed”) were positively correlated with liking, while the negative emotions (e.g., “displeased”, “sadness”, “sleepy”) were negatively related. Nonetheless, from the 15 self-reported emotions, only the scores of the emotional term “curious” were significantly different among age–gender groups when data from both wine types were considered (Table 4). In this case, the senior females provided the lowest scores for this term (Figure 2). Additionally, a significant effect (*p* < 0.05) of the type of wine (red or white) was found for “nostalgic” and “refreshed” emotional terms.

### 3.4. Relationship between Salivary Parameters and Sensory Perception

A Pearson’s correlation analysis was applied to relate salivary parameters (TPC and salivary flow rate) with aroma intensity and liking. These results are shown in Table 5.

There was not a significant correlation among salivary parameters with aroma intensity and liking for the white wine (Table 5). However, it is noteworthy that in the case of the red wine, a significant and positive correlation of salivary flow rate and black pepper intensity (0.218) and TPC and liking (0.211) was found.

## 4. Discussion

The objective of this research was to study the combined effect of age and gender on saliva composition (TPC and saliva flow rate), on retronasal aroma perception and on the hedonic and emotional response of consumers to wine. Total protein content and salivary flow rate were selected since they have been previously correlated with retronasal aroma perception during wine tasting using a Time–Intensity sensory approach [13].

Significant differences in the salivary flow rate among age–gender groups were found (Table 1). The group of younger consumers (18–35 years old) showed 14.4% higher salivary flow rate than the group of senior consumers (>55 years old). Moreover, salivary flow rate in young–females was significantly higher (13.4%) than senior–female, and the salivary flow rate in young–male was 15.3% higher than senior–male. These results correspond with previously published works. Smith and collaborators [34] observed a reduction in salivary flow rate in individuals over 70 years old when compared to 20–30 years old and 40–50 years old. Other authors [7] also proved a decrease in salivary flow rate with age, explained by a reduction in the secretion of saliva by the salivary glands. Additionally, a higher salivary flow rate in males (0.1–0.3 mL/min) than in females when comparing the same age group was also described by Smith and collaborators [34], which has been related to differences in salivary gland size and hormonal regulation or body weight profile [34,35,36].

Significantly higher TPC values were found in seniors than in young consumers (Table 1). This is also in agreement with previous studies [13,37,38]. In general, as salivary flow rate decreases, an increased concentration of proteins is expected [39,40].

Besides differences in TPC, the differences in aroma perception, liking and emotions by age–gender group were also investigated by focusing on the characteristic aroma descriptors of the wines selected for this study, i.e., black pepper note in the red wine and pineapple aroma in the white wine. These attributes were selected in previous laboratory trials as the most relevant in the two wine types. Firstly, the ability of consumers to differentiate the selected descriptors in each wine type was checked. The scores associated with the congruent red wine descriptor—black pepper—were higher in red wine than in white wine, and a similar situation accounted for the pineapple aroma. The ratings of this attribute were higher in the white than in the red wine (Figure 1). This showed that the selected aroma attributes were well-suited for the purpose of this work.

Significant differences between age–gender groups were only observed in the red wine for the black pepper aroma descriptor (Figure 1A). The largest differences in aroma perception were observed for the female consumers. The young–female group provided the highest intensity scores (4.98), while the senior females displayed the opposite, and the perceived intensity of black pepper was the lowest (2.46) of the four age–gender groups. There were not any significant differences between young– or senior–male consumers in the perceived intensity of this aroma descriptor.

Previous studies have reported that females have a better ability to perceive aromas than males, since females have a greater number of cells in the olfactory bulb [41]. Additionally, this performance seems to depend on the type and quality of the aromatic stimulus. For instance, it has been observed that females can identify the fruity, spicy, herbaceous, floral and buttery aromas more easily than males [41].

Using different olfaction techniques, Sorokowski and collaborators [42] attempted to elucidate the reasons behind the possible female superiority in olfactory perception, showing that it could be associated with neuroendocrine agents and complex interactions between hormones and the olfactory system [43,44]. Previous studies [45,46] also indicated that differences in olfaction between sexes can be related to the presence of estrogen, one of the hormones associated to increased olfactory sensitivity in females. A low quantity of circulating estrogen might compromise the olfactory abilities of older females.

Despite the differences in aroma perception by age–gender groups, no significant differences in liking were found (Table 2). This could be due to the high quality of these wines (both wines belonged to the category of “Premium wines”), or to the relevant impact of other sensory modalities in wine liking (astringency, mouthfeel, intensity, body, structure) that were not considered in this study.

Since the hedonic response might not be sufficient to explain the consumers’ response to food products [20,22,47,48], the difference among age–gender groups in the emotional response elicited during the consumption of both types of wines was also assessed. These differences were, however, not significant. The limited number of consumers in each group, due to the constraints imposed for collection and analysis of all the saliva samples, may have affected these results.

Nonetheless, from the fifteen emotional terms, age–gender differences were significant for the term “curious” (Table 4), which was higher scored by young–females, while the lowest scores were given by the senior–female group. Therefore, this emotional category was significantly different in both female groups. Previous studies [17] using different wine types also showed that the emotional term “curious” was differently rated depending on the consumers’ age. Nonetheless, in this previous study performed by Mora and collaborators [17] this term was rated higher by the senior group (over 55 years old) independently of the sex.

Previous works showed that salivary flow rate and total protein content were the parameters that most affected wine retronasal aroma perception [13,49]. However, the effect of differences in salivary composition and flow rate on aroma perception and liking using wine consumers remained unknown. Therefore, in the last step of the work, a correlation analysis using data collected from saliva composition (salivary flow rate and total protein content) and sensory analysis (perceived aroma intensity and liking) was performed. Results from the Pearson correlation analysis (Table 5) only showed significant correlations between saliva composition and sensory parameters in the case of black pepper aroma in red wine, but no correlation was found with the white wine. As shown in Table 5, salivary flow rate was significantly and positively correlated with perceived intensity of black pepper aroma, while salivary total protein content was also positively correlated with liking.

As described by Repoux and collaborators [50], salivary flow rate can affect various mechanisms involved in retronasal aroma release, and therefore it can modulate aroma intensity. For instance, during consumption, wine aroma compounds are released into the saliva and then into the air phase [50,51,52]. Additionally, aroma molecules can be adsorbed onto the mucosal pellicle that covers the oral mucosa [53]. An increased saliva flow rate could lead to a greater number of swallowing episodes, which would boost the release of a high quantity of aroma compounds travelling to the olfactory receptors. In fact, Criado and collaborators [13] also showed a high intensity perception of smoked and black pepper wine aromas in a group of senior trained panelists compared to a younger group, which was related to their differences in salivary flow rate. On the other hand, as it has been shown in the present study (Table 1), a higher salivary flow rate was generally associated with a low total protein content in saliva [13]. As the quantity of total salivary proteins decreases, the lower the number of aroma–protein binding associations or aroma metabolism by salivary enzymes will be. The consequence could be a high quantity of aroma molecules available to reach the olfactory bulb and, more likely, a high retronasal aroma perception.

This could explain why in the present work the individuals with a high salivary flow rate showed a high retronasal aroma perception. In fact, the highest intensity scores for black pepper aroma were determined in the young–female, young–male and senior–male groups, which also showed the highest salivary flow values (Figure 1A). Nonetheless, although the linear correlation found was significant, the strength of the association was weak (0.218), highlighting that besides saliva flow rate and composition, other factors (e.g., differences in olfactory performance) could also contribute to explaining these results.

Despite this, it is interesting to note that the correlation between aroma and salivary flow rate was only significant in the case of the red wine, and only for the black pepper descriptor. This result seems to agree with the involvement of the wine matrix composition on the interactions between aroma molecules and oral physiology (saliva) during wine tasting. In this sense, the largest differences between red and white wines are related to their different matrix composition, and specifically to the high content of phenolic compounds in red wines compared to white wines [54].

It is well documented that phenolic compounds can interact with salivary proteins, precipitating them, which is translated into a loss of lubricity in the oral cavity and the experience of an astringent sensation [55]. The formation of complexes between polyphenols, salivary proteins and aroma compounds were previously described [56]. The formation of these complexes depends on the type of polyphenol and on the physicochemical characteristics of the aroma molecule [57]. Wines spiked with different types of phenolic compounds could modify the release of aroma compounds in the mouth and the perception of certain aroma attributes [57]. This explanation might support the results of the present work, in which it was confirmed that saliva protein has a higher impact on the aroma perception of red wines (with phenolic compounds able to react with saliva proteins to bind aroma molecules) compared to white wines. This also might explain why the differences by age–gender groups (with different saliva protein content) on aroma perception were only noticed in red wine, but not in white wine. Even considering the same aroma attribute, likely associated with the same volatile chemical compound/s, the differences in aroma intensity among groups of consumers were only found in the case of red wine.

Having investigated differences in saliva composition (TPC) and flow rate by age–gender group, in the second part of the work we looked for differences in aroma perception, liking and emotions. The characteristic aroma descriptors of the wines selected for this study, i.e., black pepper note in the red wine and pineapple aroma in the white wine, were evaluated. This could be related to the role of salivary proteins on the astringent sensation [58,59]. Previous works showed a negative correlation between acceptability and wine astringency [15,60]. It was shown in animal studies that salivary proteins have the potential to alter the oral sensory perception of foods [61]. Although in this study only the total protein content and not the specific proteins involved in wine astringency were determined, the effect of salivary proteins on the astringency perception and therefore on wine acceptability could be hypothesized. For instance, a high proportion of salivary proteins (likely those involved in astringency) could imply that under the presence of certain types of red wine phenolic compounds there are sufficient quantities of saliva proteins to maintain a higher oral lubricity, which could be reported as a lower astringency and a high acceptability [62]. Although in this work astringency was not evaluated and only TPC were quantified, this is a plausible and interesting hypothesis to explore in future studies.

## 5. Conclusions

This study shows that differences in biological factors such as age affected salivary flow rate and total protein content as well as the black pepper and pineapple retronasal aroma perception in wine consumers. These differences were only significant in the case of red wines. Younger females perceived higher black pepper aroma intensity compared to older females. Correlation analysis confirmed the relationship between salivary flow rate and black pepper aroma perception, but only in red wines. In red wine, a positive correlation among the salivary protein content and wine acceptability was also found. These results remark the impact of oral physiology, i.e., salivary flow rate and total protein content, in retronasal aroma perception considering wine consumers. Additionally, results suggest the large involvement of wine matrix composition (phenolic compounds) on the interactions that aroma molecules initiate in the mouth during the oral processing of wine that can affect not only aroma perception, but also consumer’s hedonic response.

## Figures and Tables

**Table 1:** ANOVA and Tukey test results of saliva characterization by age–gender groups.

Age–Gender Group	Salivary Flow Rate (mL/min)	TPC (mg/L)
Senior–Male	1.33 ± 0.52 ^ab^	1448.35 ± 549.03 ^a^
Young–Male	1.57 ± 0.52 ^a^	991.39 ± 539.96 ^b^
Senior–Female	1.10 ± 0.52 ^b^	1483.44 ± 547.87 ^a^
Young–Female	1.27 ± 0.52 ^ab^	1219.75 ± 553.24 ^ab^
Pr > F	0.02	<0.01

TPC: Total protein content. Different superscripts (a-b) within the same column show significant differences among age-gender groups from Tukey test (*p* < 0.05).

**Table 2:** Mean values with standard deviation of hedonic response (liking) using a 9-point scale by age–gender groups in both wines.

Age–Gender Groups	White Wine	Red Wine
Senior–Male	6.23 ± 0.39	6.45 ± 0.39
Young–Male	6.21 ± 0.38	5.96 ± 0.38
Senior–Female	6.22 ± 0.37	6.39 ± 0.39
Young–Female	6.82 ± 0.32	6.44 ± 0.32
Pr > F	0.44	0.78

**Table 3:** Pearson correlation between liking and emotional terms.

Emotional Terms	Liking
Sensitive	**0.216**
Sleepy	**−0.223**
Lucky	**0.527**
Affectionate	**0.466**
Cheerful	**0.507**
Joyful	**0.535**
Anxious	**0.535**
Nostalgic	**0.139**
Sadness	**−0.251**
Displeased	**−0.459**
Relaxed	**0.492**
Satisfied	**0.700**
Curious	**0.439**
Fun	**0.487**
Refreshed	**0.389**

Values in bold were statistically significant (*p* < 0.05).

**Table 4:** *p*-Values of the emotional terms considering age–gender effect.

Emotional Terms	*p*-Values
Sensitive	0.23
Sleepy	0.86
Lucky	0.79
Affectionate	0.55
Cheerful	0.48
Joyful	0.76
Anxious	0.96
Nostalgic	0.57
Sadness	0.39
Displeased	0.84
Relaxed	0.09
Satisfied	0.06
Curious	**0.04**
Fun	0.64
Refreshed	0.15

Values in bold were statistically significant (*p* < 0.05).

**Table 5:** Pearson correlation coefficients between salivary parameters (salivary flow rate and total protein content), retronasal aroma intensity perception (pineapple and black pepper) and liking in both wines (white and red wine).

Variables	White Wine		Red Wine	
	Salivary Flow Rate (mL/min)	TPC (mg/L)	Salivary Flow Rate (mL/min)	TPC (mg/L)
Pineapple intensity	−0.048	0.129	−0.081	−0.072
Black pepper intensity	0.089	0.005	**0.218**	0.043
Liking	−0.001	−0.006	0.072	**0.211**

TPC: total protein content. The values in bold are statistically significant (*p* < 0.05).

## Data Availability

Not applicable.

## Supplementary Materials

The following supporting information can be downloaded at: https://www.mdpi.com/article/10.3390/foods11193104/s1, Table S1: Emotion categories and feeling terms used in this study (in English and Spanish language).
