# Supplementary material for: Understanding If Differences in Salivary Flow Rate and Total Protein Content Triggered by Biological Factors (Sex and Age) Affect Aroma Perception and the Hedonic and Emotional Response of Wine Consumers
Source: Foods. 2022 Oct 5;11(19):3104. doi: 10.3390/foods11193104 (PMC9562189; doi:10.3390/foods11193104)
Supplement: Supplementary file 1 [file foods-11-03104-s001.zip › foods-1883336-SI.pdf]

Table S1: Emotion categories and feeling terms used in this study (in English and Spanish language).

Categories	Terms
SLEEPY *Adormilado*	Adormilado
AFFECTIONATE *Afectuoso*	Afectuoso/Amoroso/ Cálido/Cariñoso/ Romántico
LUCKY *Afortunado*	Afortunado/Agradecido/ Confortado
JOYFUL *Alegre*	Alegre/Contento/Feliz/ Ilusionado
CHEERFUL *Animado*	Amigable/Animado/Bien
CURIOUS *Curiosidad*	Curiosidad
DESIROUS *Deseoso*	Ansioso- deseoso/ Emocionado-entusiasmado/Excitado/ Placer/Sorprendido positivamente
DISPLEASED *Disgustado*	Asqueado/Confuso/ Desagradado/ Descontento/Disgustado/Indiferencia/raro/ Sorprendido negativamente
FUN *Divertido*	Divertido/Enérgico/ Eufórico/Fiestero/Fuerte
NOSTALGIC *Nostálgico*	Añoranza/Melancólico/ Nostálgico
REFRESHED *Refrescado*	Refrescado
RELAXED *Relajado*	Calmado/Despreocupado/Relajado/ Sereno/ Tranquilo
SATISFIED *Satisfecho*	Complacido/Satisfecho/ Seguro
SENSITIVE *Sensible*	Sensible
SADNESS *Tristeza*	Tristeza
